# Syringe service program-based telemedicine linkage to opioid use disorder treatment: protocol for the STAMINA randomized control trial

**DOI:** 10.1186/s12889-021-10669-0

**Published:** 2021-03-31

**Authors:** Dennis P. Watson, James A. Swartz, Lisa Robison-Taylor, Mary Ellen Mackesy-Amiti, Kim Erwin, Nicole Gastala, Antonio D. Jimenez, Monte D. Staton, Sarah Messmer

**Affiliations:** 1 Lighthouse Institute, Chestnut Health Systems, 221 W Walton St, Chicago, IL 60610 USA; 2 Jane Addams College of Social Work, University of Illinois Chicago, 1040 W. Harrison St, Chicago, IL 60607 USA; 3 Center for Dissemination and Implementation Science, Department of Medicine, University of Illinois College of Medicine at Chicago, 818 S Wolcott Ave, Chicago, IL 60612 USA; 4 Division of Community Health Sciences, School of Public Health, University of Illinois at Chicago, 1603 W. Taylor St, Chicago, IL 60612 USA; 5 Institute for Healthcare Delivery Design, University of Illinois at Chicago, 1220 S. Wood Street, Chicago, IL 60612 USA; 6 Mile Square Health Centers, Department of Family Medicine, University of Illinois College of Medicine at Chicago, 1220 S Wood Street, Chicago, IL 60608 USA; 7 Community Outreach Intervention Projects, University of Illinois – Chicago, School of Public Health, 1603 W. Taylor, Chicago, IL 60612 USA; 8 Center for Dissemination and Implementation Science, Department of Medicine, University of Illinois College of Medicine at Chicago, 818 S Wolcott Ave, Chicago, IL 60612 USA; 9 Departments of Academic Internal Medicine and Pediatrics, University of Illinois Chicago, 840 S Wood St, Chicago, IL 60612 USA

**Keywords:** Telehealth, Syringe exchange, Medication assisted treatment, Medication for opioid use disorder, Substance use disorder

## Abstract

**Background:**

A key strategy for mitigating the current opioid epidemic is expanded access to medications for treating opioid use disorder (MOUD). However, interventions developed to expand MOUD access have limited ability to engage opioid users at higher levels of overdose risk, such as those who inject opioids. This paper describes the study protocol for testing STAMINA (Syringe Service Telemedicine Access for Medication-assisted Intervention through NAvigation), an intervention that engages high-risk opioid users at community-based syringe service programs (SSP) and quickly links them to MOUD using a telemedicine platform.

**Methods:**

This randomized control trial will be conducted at three SSP sites in Chicago. All participants will complete an initial assessment with a provider from a Federally Qualified Health Center who can prescribe or refer MOUD services as appropriate. The control arm will receive standard referral to treatment and the intervention arm will receive immediate telemedicine linkage to the provider and (depending on the type of MOUD prescribed) provided transportation to pick up their induction prescription (for buprenorphine or naltrexone) or attend their intake appointment (for methadone). We aim to recruit a total of 273 participants over two years to provide enough power to detect a difference in our primary outcome of MOUD treatment linkage. Secondary outcomes include treatment engagement, treatment retention, and non-MOUD opioid use. Data will be collected using structured interviews and saliva drug tests delivered at baseline, three months, and six months. Fixed and mixed effects generalized linear regression analyses and survival analysis will be conducted to compare the probabilities of a successful treatment linkage between the two arms, days retained in treatment, and post-baseline opioid and other drug use.

**Discussion:**

If successful, STAMINA’s telemedicine approach will significantly reduce the amount of time between SSP clients’ initial indication of interest in the medication and treatment initiation. Facilitating this process will likely lead to stronger additional treatment- and recovery-oriented outcomes. This study is also timely given the need for more rigorous testing of telemedicine interventions in light of temporary regulatory changes that have occurred during the COVID-19 pandemic.

**Trial registration:**

ClinicalTrials.gov (Clinical Trials ID: NCT04575324 and Protocol Number: 1138–0420). Registered 29 September 2020. The study protocol is also registered on the Open Science Framework (DOI 10.17605/OSF.IO/4853 M).

## Background

The United States has been battling an opioid epidemic for more than 20 years. The crisis, initially fueled by overprescribing, has been increasingly driven by illicitly-obtained synthetic opioids (primarily fentanyl) since 2015 [1]. A key strategy for mitigating the epidemic is expanded access to medications for treating opioid use disorder (MOUD), which include methadone, buprenorphine, and injectable naltrexone. When used appropriately, all three medications are demonstrated to be superior to alternative, non-medication-based treatments in improving outcomes (e.g., opioid cravings and use, overdose, and resulting mortality) for people living with opioid use disorder (OUD) [2–5]. However, interventions developed to expand MOUD access, while beneficial, remain limited in their ability to engage opioid users at higher levels of overdose risk, such as those who inject. This is because such interventions often require individuals to initiate treatment seeking from a healthcare system they might not fully trust [6], or they rely on emergency medical services as a mechanism of post-overdose engagement [7–10]. One possible solution is to develop interventions that engage high-risk opioid users in trusted settings and link them to MOUD as rapidly as possible after they indicate interest in treatment. STAMINA (Syringe Service Telemedicine Access for Medication-assisted Intervention through NAvigation) is an intervention developed to address these issues by engaging individuals at a community-based syringe service program (SSP) and then quickly linking them to MOUD using a telemedicine platform. This paper describes the study protocol that aims to assess the effectiveness of STAMINA as an MOUD treatment linkage intervention.

The STAMINA intervention is located in an SSP setting where the primary purpose is providing free access to sterile syringes for people who inject drugs [11]. Ancillary services also provided at the SSP include linkage to medical care and substance use disorder treatment. The rationale for locating STAMINA in an SSP is because they are generally perceived to be judgment-free areas where users of illicit drugs feel more comfortable accessing services than they might in other healthcare settings. For instance, previous research has demonstrated SSP clients are comfortable with and trusting of SSP staff and the information provided to them [6, 12–15]. Client acceptability and satisfaction with SSP services are likely driven by the harm reduction approach employed, which emphasizes low-barrier services aimed at reducing risks associated with drug use, while offering (but not requiring) connection to formal treatment and healthcare services [16]. Additionally, a vast array of studies have demonstrated SSP services can improve outcomes specifically for injection drug users. Most notably, SSPs are demonstrated to reduce risky injection behaviors [11, 17–19] and reduce HIV transmission [17, 20, 21]. Additional studies have demonstrated enhanced SSP-related benefits when services are combined with MOUD and other types of substance use disorder treatment [11, 19, 22]. However, while SSP clients often express interest in treatment, thereby affording a potentially important opportunity for treatment linkage [23–25], assessments of the actual engagement rates have ranged from 5 to 70%; with neither the factors underlying this variability nor the optimal way to facilitate treatment linkage being well understood [11, 24, 26–35].

STAMINA comprises five primary components. First, a private physical space in the SSP is used to carry out intervention procedures. Second, staff conduct a brief interview to assess MOUD interest. Third is a same-day secure, video-based telemedicine appointment with a provider who can offer treatment (either directly or through referral) with all three MOUD types. Fourth, rideshare-based transportation assistance is provided to each participant at one time point depending on the MOUD prescribed by the telemedicine provider: (a) for participants determined to be best treated with buprenorphine or naltrexone, an immediate rideshare to the pharmacy is offered to facilitate pick up the induction dose of their medication and (b) for participants prescribed methadone, a rideshare is offered for their scheduled intake appointment. Fifth, is the ability to cover the cost of the induction medication for those prescribe buprenorphine or naltrexone who do not have another payment source. As a linkage-only intervention, STAMINA does not provide any ongoing supports, which are instead provided by SSP staff and/or the treatment provider.

A handful of prior randomized control trials have demonstrated mixed results related to SSP-based OUD treatment linkage interventions. For instance, three separate trials have tested treatment referral with case management services against standard referral [26, 34, 36]; however, only one by Strathdee et al. [34] saw improved outcomes in the experimental arm. Kidorf and colleagues [28, 30, 37] have conducted three trials comparing various monetary incentive-based approaches to other linkage models (e.g., motivation sessions, low threshold services, and standard referral). Two of these trials [28, 30] demonstrated improved linkage or engagement for those who received monetary incentives, and the third [37] showed no difference between groups.

There are a number of differences among these previously tested interventions that might account for their variable performance. For instance, time between intervention start and treatment linkage can be up to 7 days [26, 34]—shortening this window is likely to improve patient follow-through with referral [38, 39]. Additionally, many prior studies mandated counseling or motivational sessions as part of the tested intervention or as part of general research participation [28, 30, 37]. However, requiring engagement in psychosocial services has been noted as a potential barrier to MOUD participation [40]. STAMINA addresses these weaknesses using a telemedicine model for same-day treatment linkage and medication induction without additional service requirements.

The STAMINA trial will also provide much needed knowledge related to the telemedicine approach. While, embedding and MOUD provider at an SSP would hypothetically result in similar reductions in linkage and medication induction times as telemedicine, there is limited research regarding such approaches [41]. Moreover, telemedicine is potentially more feasible to implement, cost effective, and sustainable for SSP settings. However, there have been considerable barriers to telemedicine implementation for MOUD (e.g., lack of reimbursement, use restricted to largely rural populations, staffing requirements, prior in-person examination requirements, and types of medications that can be prescribed) that have prevented its widespread use [42, 43]. This is unfortunate, as prior research has shown telehealth interventions—which include telemedicine—to be associated with higher patient satisfaction and equal effectiveness when compared to in-person treatment for OUD and other substance use disorders [44]. Recently, the coronavirus disease 2019 (COVID-19) pandemic has led to relaxation in federal guidelines for telemedicine-based MOUD treatment that has led to wider implementation [45, 46]. However, these changes were originally set to expire in December of 2020 and have not been made permanent at this time this manuscript was developed. As such, there is a need for both observational studies related to these policy changes and rigorous clinical trial knowledge to assess the benefits of telemedicine for OUD treatment.

The STAMINA trial will follow a parallel randomized design. The primary question guiding the trial is whether the STAMINA intervention improves MOUD linkage above referral as usual. Additionally, there is a goal to understand the potential effect of STAMINA on MOUD engagement and retention and illicit opioid use. The primary hypothesis is that (1) STAMINA will be more effective at improving MOUD linkage than standard referral. Secondary hypotheses are: (2) the STAMINA participants will have greater MOUD engagement than participants who receive standard referral; (3) STAMINA participants will have greater MOUD retention than participants who receive standard referral; (4) STAMINA participants will have used less illicit opioids at follow-up than participants who received standard referral.

## Methods^1^

### Study setting

The study will be conducted at three Chicago-based and university-affiliated SSP sites. The sites are located in different areas of the city and serve demographically diverse local populations. For example, the SSPs on Chicago’s west and south sides serve primarily an African American/Black clientele, 83.0 and 93.2% respectively, whereas the northwest side office serves a higher percentage of Latinx clients (52.0%). To assure the aggregate study sample captures both the geographical as well as racial/ethnic diversity of SSP clients, the study will recruit and enroll participants at each of the locations. Initial MOUD treatment assessment will be provided by a university-affiliated Federally Qualified Health Center (FQHC) that can prescribe buprenorphine and naltrexone and has a relationship with a methadone treatment provider to whom patients can be referred as appropriate. Trial participants prescribed buprenorphine or naltrexone can receive MOUD treatment at any of the 6 FQHC locations across the city, three of which are in close proximity to SSP sites. Additionally, induction buprenorphine and naloxone prescriptions can be dispensed from the main FQHC office at no cost to study participants who lack a payment source. The FQHC can also refer to methadone clinics in various locations throughout the city.

### Potential participants

A power analysis determined a minimum sample size of *n* = 273 is needed to detect an odds ratio of 2.0 for a binary predictor (the intervention effect) in a logistic regression with 80% power. The control group’s proportion of successful treatment initiation was set at 0.35. Assuming an 80% study retention rate across all three waves of data collection, the recruitment goal has been set at *n* = 350 total participants (*n* = 175 for each arm).

To be included in the study, potential participants must: communicate verbally in English, be at least 18 years of age, reside in Cook County, Illinois, meet clinical criteria for a past-year opioid use disorder of any severity level [47], and express interest in receiving MOUD treatment. Exclusion criteria include: having plans to move outside of Cook County, Illinois within the next six months; under criminal justice supervision that requires serving a jail or prison sentence within the next six months; experiencing severe withdrawal symptoms as indicated by the Clinical Opiate Withdrawal Scale [48]; currently taking any medication prescribed to them by a healthcare provider to treat OUD; or demonstrating inadequate ability to provide informed consent.

### Study arms and allocation process

Participants will be assigned to one of two study arms using randomization with stratification by site and whether or not they indicate a strong preference for methadone treatment. The reason for stratifying assignment on this second condition is to avoid potential confounding of the results since methadone treatment will be administered by a different provider and (as described below) require different linkage procedures after the telemedicine call. Allocation will be determined in advance of enrollment by randomizing pre-established study identification numbers that will be sequentially assigned to participants as they are enrolled.

Intervention arm participants will have their vitals taken and shared with a provider (a medical doctor, physician’s assistant, or nurse practitioner who is waivered to prescribe buprenorphine) at the beginning of the telemedicine video call, which will be conducted via a tablet computer. If buprenorphine or naltrexone is prescribed, the provider will schedule a follow-up visit, and the participant will be provided instructions for home induction and offered immediate rideshare transportation to the FQHC’s pharmacy. For methadone, the provider will make a referral, and the research assistant will immediately assist the participant to make an appointment and offer to arrange a rideshare for the intake visit. Researchers will also provide an immediate rideshare to the FQHC’s pharmacy if the provider prescribes medication to assist with any withdrawal symptoms the patient might experience until the methadone intake appointment. Participants assigned to the control arm will be given a standard referral for an in-person intake appointment at an FQHC location or methadone clinic, and researchers will make every attempt to ensure thee appointments occur within 24–72 h of enrollment. Control participants will also be provided with two one-way bus passes if transportation assistance is needed. Patients in both arms will have access to all services offered by the FQHC or methadone clinic, and continuing treatment for both arms will be provided following the organization’s (e.g., FQHC or methadone clinic) general standard of care.

Though unlikely given the population, it is possible a provider could assess a participant from either arm as being inappropriate for MOUD treatment. If this occurs, the provider’s decision will be noted, and the patient will continue to be followed up with for the normal study duration.

### Measures

Basic demographic information on all participants will be collected at baseline, as well as data reflecting social support, child welfare involvement, alcohol use, opioid cravings, overdose experiences, past-year and past-month drug use and treatment, chronic health conditions (see [49]), psychological health (see [50]), trauma exposure, and patient comfort with telemedicine or in-person MOUD intake.

The primary outcome is linkage to MOUD treatment, which is defined as whether or not the participant attends one MOUD treatment appointment within 14 days of study enrollment [49] and measured using administrative FQHC and methadone provider treatment data. As the intervention group would automatically meet this outcome after their initial telemedicine call, only those appointments made after participants leave the enrollment site will be counted. There are three secondary outcome measures: (1) MOUD treatment engagement, defined as attending a minimum of two OUD-related treatment appointments within 34 days of study enrollment [51] will measured using administrative data from providers that tracks reasons for the treatment appointment and whether they were kept or not. (2) MOUD treatment retention will also be measured using administrative data and defined as the number of days the patient is actively engaged in treatment, with active engagement being defined as (a) not being discharged from care or (b) going no more than 14 days without medication. The operationalization for this outcome will differ depending on the MOUD: 15 or more days without renewing a buprenorphine prescription, missing 15 or more consecutive methadone doses, or 45 or more days since the last naltrexone injection. Participants will still be considered engaged if they switch to a different MOUD with the 14-day window. (3) Non-MOUD opioid use will be measured in two ways: (a) self-reported days of non-prescribed/medically supervised opioid use in the past 30 days using questions from the *National Survey on Drug Use and Health* [52] modified to ask about separate known use of heroin, fentanyl/carfentanil, and prescription painkillers; and (b) detection of illicit opioids through a saliva test administered at the time of data collection. Self-reported treatment linkage and engagement data for any care received from outside of the study’s treatment partners will be collected (in addition to the FQHC’s administrative records) to understand if participants engaged in treatment with another provider. In addition to measuring these outcomes, additional information will also be collected related to: all cause and opioid-related mortality from state vital records, self-reported criminal justice involvement, and health-related quality of life using the current quality of life scale [53].

### Procedures

Table 1 displays the expected participant timeline for the study. Recruitment will occur at SSP locations. Potential trial participants will be identified one of two ways: (1) they will see recruitment materials posted and request more information from SSP staff or (2) SSP staff will inform clients who indicate treatment interest of the opportunity to participate in the trial. SSP staff will then refer interested individuals to research staff. Research staff will provide more detailed information, conduct an eligibility assessment based on the previously listed inclusion and exclusion criteria, and complete the initial consent process with those deemed eligible to participate.

**Table 1:** Participant timeline

	Study Period				
	Enrollment	Allocation	Post-allocation		Close-out
Time point	-t_1_	0	t_1 (baseline)_	t_2 (3 months)_	t_3 (6 months)_
ENROLLMENT:					
Eligibility assessment	x				
Informed consent	x				
Allocation		x			
DATA COLLECTION:					
Baseline characteristics			x		
Telemedicine preference			x		
Treatment linkage, engagement, & retention (FQHC records)				x	x
Opioid use (self-report)			x	x	x
Criminal justice involvement (self-report)			x	x	x
Quality of life (self-report)			x	x	x
Mortality (state vital records)				x	x
Drug test			x	x	x
INTERVENTION:					
Telemedicine Linkage			x		
Standard referral			x		

After consent is obtained, research staff will collect baseline data using a structured questionnaire and administer a 14-panel saliva drug test. After all data are collected, research staff will then open a sealed envelope containing a card informing them of the trial arm to which the participant’s sequential study identification number has been assigned. Depending on the arm assignment, the participant will then receive a telemedicine visit or standard referral.

Participants will be asked to return to the SSP for three- and six-month follow-up data collection. On-site research staff will administer the saliva test and connect them by phone to off-site research staff who will not know the participant’s arm assignment to complete the structured questionnaire. Participants will receive a $25 incentive for completing baseline data collection. At follow-up interviews, they will receive $35 (to accommodate the additional travel time to and from the interviews) and will also be offered public transportation passes to accommodate any travel expenses accrued.

Following procedures outlined by Scott et al. [54], research personnel will utilize the client contact information obtained during the baseline interview to locate and retain participants for follow-up data collection activities. Approximately one week following baseline, research personnel will attempt to verify all contact information provided by the participant, including the contact information for their friends, family, and other associates. One month in advance, research personnel will attempt to contact the participant to schedule each follow-up. If research staff are unable to contact the participant directly, the contact information for friends, family, or other associates will be utilized.

To protect participant confidentiality, all data will be stored in the HIPAA-compliant Research Electronic Data Capture (REDCap) system [55]. Only limited members of the research team will have access to this REDCap project via their personal, password protected login information. Further, study identification numbers will only be connected to participants’ names in REDCap. A secure Box Health account will be used to store data obtained from participant records, which will be shared using a secure transfer process. All completed informed consent documents will be stored in a secure and locked location. A certificate of confidentiality (CC-OD-20-900) has been received from the National Institute on Drug Abuse to provide an added layer of legal protections for participants’ data. Finally, if researchers must reach out to identified contacts to locate participants, they will only inform the contact that the participant is in a research study for which particular details cannot be discussed.

All protocols are outlined in the study’s protocol and procedures manual. All research assistants must read this manual, role play completing the interviews from both the research assistant and participant perspectives, complete two full mock enrollments, and competently carry out an observed enrollment of a true participant before they can work alone. Research assistants float among the data collection sites, rather than being assigned to a particular one. The project manager uses a checklist to complete at least one fidelity review a month with each research assistant, and their performance is reviewed with them.

### Data analysis

To assess the first hypothesis, multivariable, binary logistic regression models will be used with linkage (yes/no) as the dependent variable. The primary independent variable in these models will be a dichotomous indicator for treatment condition (0 = control arm; 1 = intervention arm). Possible additional predictors to assess the effects on outcomes as well as potential interaction effects will include, at a minimum: demographics (age, sex, race/ethnicity), employment status, baseline severity of substance use disorder, baseline severity of psychological distress, withdrawal severity at enrollment, any homelessness in the past three months (dichotomous) number of past overdose experiences, and whether treatment assignment matched expressed treatment preference at baseline. The effects of SSP location will also be measured as a trichotomous variable to represent each of the three COIP sites. Finally, as there could be differences in the primary outcome owing to the MOUD treatment participants receive, we plan on including treatment assignment as an additional model predictor. We can then test if there is an interaction between MOUD received and treatment condition. As indicated above, the study is powered to detect a two-fold increase in the odds of treatment initiation for participants in the treatment condition relative to the odds of linkage for participants in the control/standard referral condition.

Regarding the other three hypotheses, secondary outcomes such as treatment engagement, treatment retention, and past 30-day opioid use can be modeled using generalized linear mixed effects models with random intercept terms included for participants, reflecting the repeated measures nature of the study data. The type of regression model will be selected based on the expected distribution of the dependent variable (e.g., binary logistic for dichotomous dependent variables; and Poisson or negative binomial regressions for count data). An alternate measure of retention is to assess how long each participant remained in treatment defined as being either the last treatment appointment kept, or participant’s self-report of the date they discontinued using the MOUD prescribed. The time to discontinuation of treatment/medication can then be modeled using survival analysis or proportional hazard models. These models provide information on how long persons remain in treatment allowing comparisons of the survival times (or conversely, periods of highest risk for dropping out of treatment) by study condition as well as other covariates.

The effects of missing data on the analysis will be handled according to the amount of missing data. If only a small proportion (< 5%) of cases have missing data on the dependent variable and model predictors, simple listwise deletion will be used [56]. If there is a larger proportion of cases with missing data, the pattern of missingness on model covariates will first be examined to determine if any particular covariate is disproportionately contributing to the missing data. By creating a dichotomous indicator for each covariate to indicate missing or not, preliminary bivariate regression models can be run for each dependent variable to determine if missing cases significantly differ from non-missing cases related to the outcomes of focus. The models will then be estimated using listwise deletion. Next, the models will be re-estimated using full information maximum likelihood within a structural equation or a generalized structural equation framework [57, 58]. Comparison of the two sets of results will provide a sensitivity analysis of the effects of missing data on parameter estimates.

### Data safety and monitoring

The Principal Investigators (DPW and JAS) will be responsible for all data oversite. Data will be reviewed for quality as it is acquired, and, any problems identified will be noted and feedback will be provided to research staff. Prior to each quarterly Data Safety and Monitoring Board (DSMB) meeting, an analysis will be conducted of existing data to identify potential negative trends. All significant negative trends and adverse events will be discussed in detail with the DSMB (comprising three individuals with randomized control trial, substance use disorder, syringe exchange populations, and MOUD treatment among them) to assess whether they resulted from involvement in the study or other possible factors. If a link between study involvement and negative trends or adverse events is established, a review will be conducted to understand whether these differences are related to amenable study procedures. If negative trends are demonstrated to be related to the study, data collection will be stopped. Subject withdrawals and any complaints will be monitored to ensure study procedures designed to protect the privacy and confidentiality of participants are adequate and no unanticipated distress or unintended outcomes have resulted from any of the study procedures. In addition, the institutional review board and DSMB will be notified within 24 h of any unexpected and serious adverse events that could potentially be related to study involvement, and the researchers will conduct a review of study protocols and procedures to identify corrective measures.

### Ancillary and post-trial care

After the end of the study, trial participants will be able to continue receiving services at the provider they are receiving treatment from and the SSP indefinitely.

### COVID-19 considerations

COVID-19 is having direct impacts on opioid users that need to be considered and accounted for during the trial’s course. For instance, social distancing can result in isolation that cuts people off from the supports that are an important component of recovery [59, 60]. People who use opioids are also at greater risk of complications and death if they do catch the virus [60]. The temporary relaxation of telemedicine guidelines has changed treatment standards since this project was initially conceived, and this might impact the ability to see a difference in secondary outcomes since telemedicine improves access and will be available to both groups for their appointments after initial MOUD linkage. Given sample size calculations were completed in a pre-COVID-19 context, assumptions underlying the power analysis will be re-checked after the first 100 participants have been enrolled, appropriate adjustments will be made. Additionally, all procedures described above will now be performed with limited interactions between participants and staff to decrease risk of COVID-19 transmission. To accomplish this, SSP staff will place participants in a room and off-site research staff will conduct data collection using video conferencing technology. Onsite research staff (wearing personal protective equipment) will be available to obtain consent, complete drug testing, take vitals, and provide the research incentive. Finally, the pandemic might result in difficulties with follow-up data collection. If follow-up rates dip below 85% in any one month, participants will be given the option to complete all data collection over the phone and saliva tests will be optional. Furthermore, the follow-up incentive will be split--$25 for the interview and $10 for the saliva test—to ensure those who choose to complete saliva testing are appropriately compensated for their travel time.

## Discussion

The STAMINA intervention was developed to improve MOUD treatment linkage for a highly vulnerable population of opioid users who are unlikely to engage with alternate linkage strategies that are often integrated within the traditional healthcare system and/or rely on a post overdose event as the route for identifying patients [6–10]. If successful, the telemedicine approach will significantly reduce the time between an individual’s indicating interest in initiating MOUD treatment and speaking to a provider. By facilitating and shortening this process, it is hoped participants will achieve improved treatment- and recovery-oriented outcomes (e.g., engagement, retention, reduced substance use, etc.) [38, 39].

A primary limitation of the study design is reliance on participant self-report for certain measures. While saliva tests are being in addition to self-reported substance use, the tests are largely unable to identify many drugs after 24–48 h. While urine tests have potential to identify some substances further out, the community advisory board made up of SSP staff and former and current opioid users advised that saliva testing would be considered less invasive by participants. STAMINA researchers are establishing a relationship with the clinic to which the FQHC will refer methadone participants for the purpose of obtaining treatment-related administrative data. Failure to establish this relationship will mean the only treatment data available from participants referred to the clinic will be self-reported.

Relatedly, it is possible that the partner FQHC will start providing methadone treatment during the course of the study. Implementation of their methadone program was scheduled to be completed before the trial began but was paused due to the COVID-19 pandemic. As a result, participants enrolled who express a strong or exclusive interest in methadone treatment, and do so prior to the FQHC receiving approval, will be referred to the methadone treatment provider. Participants enrolled after the FQHC is approved for methadone treatment will be referred to the FQHC. There will thus be a potential confound if there are provider differences in initiation or engagement. The methadone provider will be recorded as a variable, allowing statistical control for program effects, but random assignment to a methadone provider is not possible.

Possible provider effects are another limitation. However, the same providers deliver both telemedicine and in-person treatment, and this reduces the likelihood of provider effects confounding results between the two treatment conditions. Furthermore, we will be able to track which providers patients see, which will allow us to investigate possible confounding by capturing provider effects as a dummy-coded fixed effect in the regression models to test if there are any differential outcomes by assigned provider.

Finally, the data collected in the trial is limited in its ability to describe in any detailed fashion participants’ experiences with the different types of treatment, which could be beneficial for explaining trial outcomes and making improvements for future research and replication. To address this limitation, we plan to conduct a nested qualitative study [61]. However, the exact questions guiding this effort will be informed by formative trial results identified after the first 100 participants complete their first 3-month follow-up.

Interventions are needed that can identify the most vulnerable users of opioids and rapidly connect them to treatment. The STAMINA intervention attempts to fill this gap using an SSP-based telemedicine linkage model. Should STAMINA prove to be a stronger alternative to referral as usual, next steps will be to understand the intervention’s cost effectiveness and how it might be replicated in other areas despite recognized issues such as limited SSP coverage, wide ranging variations in SSP service quality, and “not in my backyard attitudes” that are likely barriers to implementation [62–64].

## Data Availability

Due to the sensitivity of information related to substance use and treatment, the dataset will only be available upon request and with proper data use agreements in place. All materials developed for the study are available to be shared upon request.

## Abbreviations

COVID-19: Coronavirus disease 2019
DSMB: Data safety and monitoring board
FQHC: Federally Qualified Health Center
MOUD: Medications for opioid use disorder
OUD: Opioid use disorder
SSP: Syringe service program
STAMINA: Syringe Service Telemedicine Access for Medication-assisted Intervention through Navigation

## Footnotes

1 Trial status: Data collection for this trial started on August 24th, 2020 at the lowest volume site and was paused due to the implementation of new administrative rules regarding COVID-19 and study safety. As such, 5 participants have been enrolled at the time the manuscript was originally submitted for peer review on October 28, 2020. The other two sites are scheduled to go live in early to mid-November 2020.
